# Pilulæ Metalorum Et Amarum

**Published:** 1867-07

**Authors:** 


Pilulæ Metalorum et Amarum,—These pills "of the bitters and metals" were invented (?) by Dr. Peake, of California, though many physicians have long been in the habit of combining about all valuable remedies of the materia medica in some one prescription. Since the publication of Dr. Peake's formula and recommendations, published in the January *Journal of Pharmacy* for 1867, Mr. Peabody, druggist in Buffalo, has had a quanity of these pills made and coated with sugar. The following is the original formula:

℞ Quiniæ sulphatis, ʒj.

Ferri Redacti, ʒjss.

Strychniæ.

Acidi Arseniosi, aa grs. iij.

Confectionis Rosarum.

Vel Mucilaginis Acaciæ.

q. s. ut ft. pil. lx.

The author says: "I would do a sort of shot-gun practice, and combine the whole of these drugs in appropriate proportions."

